# Stricture Prevention after Extensive Endoscopic Submucosal Dissection of Neoplastic Barrett's Esophagus: Individualized Oral Steroid Prophylaxis

**DOI:** 10.1155/2019/2075256

**Published:** 2019-04-14

**Authors:** Andreas Probst, Alanna Ebigbo, Bruno Märkl, Tina Schaller, Matthias Anthuber, Helmut Messmann

**Affiliations:** ^1^Department of Gastroenterology, Universitätsklinikum Augsburg, 86156 Augsburg, Germany; ^2^Institute of Pathology, Universitätsklinikum Augsburg, 86156 Augsburg, Germany; ^3^Department of General, Visceral and Transplantation Surgery, Universitätsklinikum Augsburg, 86156 Augsburg, Germany

## Abstract

**Introduction:**

Endoscopic resection (ER) exceeding ≥75% of the esophageal circumference is accompanied with a high stricture risk regardless of the resection method. The ideal strategy for stricture prevention is not well defined today. Different approaches have been reported but data are limited to the resection of squamous cell neoplasia. The aim of this study was to assess the efficacy of an individualized oral steroid regimen to prevent strictures after extensive ER in neoplastic Barrett's esophagus (NBE).

**Materials and Methods:**

Over a 50-month period, endoscopic submucosal dissection (ESD) was performed in 193 patients with NBE. 23 patients with resections exceeding 75% of the circumference were included. 19 resection ulcers were noncircumferential (NCR) while 4 were circumferential (CR). Stricture prevention was performed using oral prednisolone starting with a daily dose of 50 mg and standard tapering over 8 weeks (50/40/30/25/20/15/10/5 mg). Tapering was individualized according to the ulcer healing process (assessed endoscopically in the first tapering period and before stopping the steroids). Data were analyzed retrospectively.

**Results:**

Stricture rates were 5.3% (1/19) for NCR and 100% (4/4) for CR (*p* < 0.001). The only stricture in the NCR group was seen in a patient who had stopped steroids without any reason after few days. 12/19 patients received standard tapering over 8 weeks (63.1%). According to the individual ulcer healing, treatment was prolonged to 9-10 weeks in 4/19 (21.1%) and shortened to 7 weeks in another 2/19 (10.5%). After CR, all patients needed endoscopic balloon dilatation (median 6.5 sessions; range 3-14 sessions for 8-40 weeks). Side effects of the steroid therapy were not noted.

**Conclusion:**

Oral prednisolone therapy with an endoscopy-based individualized tapering regimen is effective in avoiding strictures after NCR of Barrett's neoplasia. After CR, the stricture risk is not sufficiently decreased. CR should be restricted to circumferential neoplasia which is a very rare scenario in neoplastic BE.

## 1. Introduction

The incidence of esophageal adenocarcinoma (EAC) is rising in Western countries [1]. Progress in endoscopic technology and surveillance programs for patients with Barrett's esophagus (BE) have improved the diagnosis of EAC in early stages allowing endoscopic resection (ER) as a curative treatment option. Endoscopic mucosal resection (EMR) of visible neoplastic lesions and additional ablation of the residual Barrett's are the standard treatment of neoplastic BE today. In selected neoplasia (lesion diameter exceeding 15 mm, poor-lifting lesions, and lesions at risk for submucosal invasion), endoscopic submucosal dissection (ESD) can be considered as a treatment option in order to achieve R0 resection and to improve histopathological assessment of R0 resection [2]. When ER is performed circumferentially or the resection area exceeds three quarters of the circumference, a substantial stricture risk has been reported for EMR (49.7-88%) and also for ESD (60.0%) in BE [3–5]. Different strategies have been introduced to prevent stricture development (balloon dilatation, stenting, local or systemic steroid therapy in fixed-dosage regimens, and tissue-shielding techniques). These techniques have been shown to reduce but not to eliminate the stricture risk, and the ideal strategy for stricture prevention remains undefined. In our previous study on ESD for neoplastic BE and early esophageal squamous cell cancers (SCCs), we performed prophylactic endoscopic balloon dilatation (EBD) in the first study period and used a fixed-dose regimen of oral steroids in the second. In the dilatation group, a high number of EBD sessions (mean 8.2) were needed to prevent strictures and perforation was noted during EBD in one patient. In the steroid group, 62.5% developed a stricture during the steroid tapering period and EBD was required also in these patients [6]. Data on stricture prevention, published mainly by Asian authors, are restricted to ER of SCCs due to the rareness of Barrett's esophagus in Asia. The aim of this study was to evaluate the efficacy of a modified stricture prevention strategy after extensive ER in neoplastic BE (oral steroid treatment regimen with endoscopy-based control of dosage and duration).

## 2. Patients and Methods

The study was conducted as a single-center uncontrolled study in a German referral center (Department of Gastroenterology, Klinikum Augsburg, Germany). All patients who underwent ESD of Barrett's neoplasia from May 2014 to July 2018 were screened. All patients had given written informed consent after receiving detailed information about the ESD procedure and alternative treatment options (EMR, surgery). Data were analyzed retrospectively. The study was approved by the Institutional Review Board of Klinikum Augsburg, Germany (IRB number BKF-A-2018-24).

### 2.1. Inclusion Criteria

Inclusion criteria are as follows:

- ESD in neoplastic BE
- Resection ulcer ≥ 75% of the esophageal circumference

- Noncircumferential resection (NCR)
- Resection involving the entire circumference (CR)

(iii) Stricture prevention performed with oral steroids

### 2.2. Exclusion Criteria

Exclusion criteria are as follows:

- Stricture prevention with other treatment regimens than oral steroids (local steroid injection into the resection ulcer, combination of oral and local steroids)
- Patients receiving steroid therapy for other indications

### 2.3. Study End Points

The primary end point was the stricture rate after ESD. Secondary end points were procedural characteristics (procedure time, R0 resection rate, curative resection rate, and other complications than stricture).

### 2.4. ESD Procedure

All patients had been referred for ER, and biopsies had shown high-grade dysplasia or EAC. Video endoscopy with white light and narrow band imaging was performed with a video gastroscope (GIF-HQ190; Olympus Medical Systems, Tokyo, Japan). When the lesion lateral margin was unclear, chromoendoscopy with acetic acid and indigo carmine was added. Lesions were classified according to the Paris classification [7]. EUS was not performed routinely. A transparent cap at the tip of the scope (D-201-11804, Olympus) and insufflation with carbon dioxide were used routinely. Resection margins were marked using the tip of a hook knife (KD-620LR; Olympus). The standard solution for submucosal injection was a mixture of saline, epinephrine (1 : 100.000), glycerol (10%), and a slight amount of indigo carmine. In cases with severe fibrosis, hyaluronic acid (Sigmavisc™, Hyaltech Ltd., Livingston, UK) was injected. A VIO 300D electrosurgical generator (ERBE Elektromedizin, Tübingen, Germany) was used (spray coag mode 25 W for marking; endo cut I mode 60−80 W for cutting and spray coag mode 60 W for coagulation during dissection). Mucosal incision and submucosal dissection were performed with the hook knife. ESD was performed under general anesthesia. Patients stayed in the hospital for 48−96 hours after ESD. Routine control endoscopies were not performed before discharge. Anticoagulants, except aspirin, had been stopped before ESD and were restarted 5-7 days after the procedure depending on endoscopist's decision.

### 2.5. Histopathologic Workup

Intramucosal lesions were classified as low-grade dysplasia (LGD), high-grade dysplasia (HGD), or mucosal cancer. Invasion depth, grading, and the presence or absence of lymphovascular invasion were described. Regarding their invasion depth, lesions were classified mucosal (pT1a) or submucosal (pT1b). Grading was categorized into G1 (well differentiated), G2 (moderately differentiated), and G3 (poorly differentiated). R0 or R1 was diagnosed for the vertical margin (VM) and the horizontal margin (HM). Curative resection was defined as R0 resection of a well- or moderately differentiated intramucosal cancer without lymphovascular invasion.

### 2.6. Complications

Stricture was defined as a complication when it was impossible to pass the esophagus using a standard gastroscope (e.g., GIF-HQ190; diameter 9.9 mm). Delayed bleeding was defined as when clinical bleeding signs were observed after ESD (hematemesis, melena, and hemoglobin drop > 2 g/dl). In these cases, endoscopic treatment was performed. Perforation was defined as an endoscopic view into the mediastinum or the peritoneal cavity.

### 2.7. Regimen for Stricture Prevention and Follow-Up

**Figure 1:**
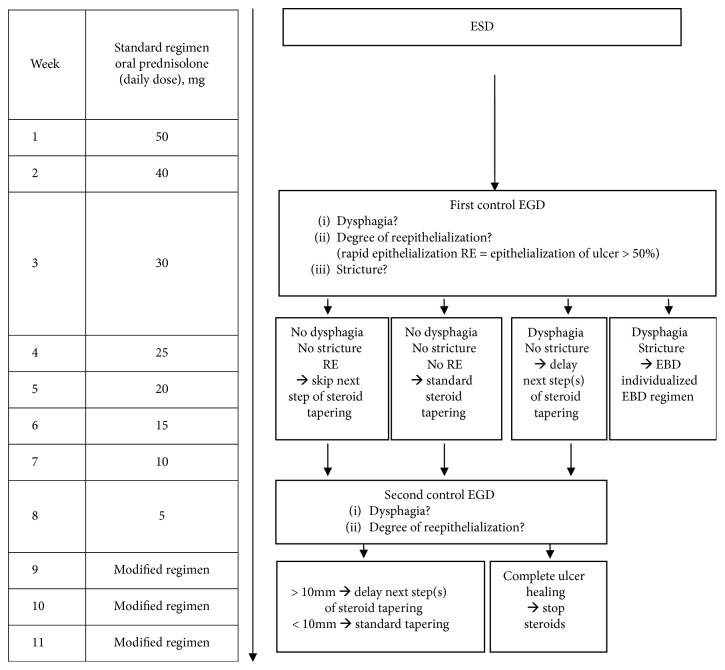
Regimen for stricture prevention (EBD: endoscopic balloon dilatation).

Based on the results of our previous study and based on the published literature on preventive steroid treatment, we developed a modified steroid-based regimen and used it from 2014 [6, 8–12]. Taking Asian data into account, we chose a starting dose of 50 mg prednisolone daily and tapered this gradually over 8 weeks (50/40/30/25/20/15/10/5 mg) resulting in a cumulative dose of 1365 mg. Prednisolone was started on the first day after ESD when the resection ulcer exceeded three quarters of the esophageal circumference. Extension of the resection ulcer was estimated at the end of the ESD procedure. We performed a first control endoscopy in the third week after ESD (days 15-22) under a daily prednisolone dose of 30 mg. Patients' symptoms, the extent of ulcer healing (reepithelialization from the ulcer margins), and the presence of stricture were assessed. Reepithelialization was defined as rapid (RE) when it exceeded 50% of the initial resection area. When patients denied dysphagia, the steroid treatment was tapered according to the degree of reepithelialization. When RE was noted, the next step of steroid tapering was skipped. When reepithelialization was not rapid, steroids were continued using the standard tapering regimen. When passage with the gastroscope was possible but patients reported any kind of dysphagia, the next step of steroid tapering was delayed for one or two weeks. When stricture had developed, EBD was started and continued according to the endoscopist's recommendation. In patients without stricture, a second control endoscopy was recommended in week eight. When complete healing of the ulcer was seen, steroids were stopped. When small residual ulcers (≤10 mm) were diagnosed, completion of the steroid treatment was recommended according to the standard tapering regimen. When large ulcers (>10 mm) were present, a daily prednisolone dose of 5 mg was recommended for another 1-2 weeks. The treatment algorithm is shown in Figure 1. When patients reported dysphagia, endoscopy was performed on demand at any time to rule out strictures. Acid suppression with proton pump inhibitors (PPI) was started at the latest on the day before ESD and was continued for three months in all patients (pantoprazole 40 mg twice daily). When residual Barrett's epithelium was seen in the second control endoscopy, patients were scheduled for ablative therapy later and PPI therapy was continued until then.

### 2.8. Statistical Analysis

Calculations were performed using the software package Sigma Plot 13.0 (Systat Software, San Jose, USA). Numeric values were compared using the Mann-Whitney test. For the comparison of categorical data, a chi-squared test was employed. *p* values <0.05 were considered statistically significant.

## 3. Results

### 3.1. Patients and Lesion Characteristics

Over a 50-month period, 193 ESD procedures were performed for neoplastic BE. 27 resection ulcers exceeded ≥75% of the circumference (13.7%). Three patients were excluded because they had received additional intralesional triamcinolone injection during the first study period. Another patient was excluded because of permanent steroid treatment performed for rheumatoid arthritis. 23 patients who started the proposed stricture prevention regimen were included for further analysis. The reason for extensive ESD was a large neoplasia in 13 patients (56.5%) and multifocal visible lesions in another 10 patients (43.5%) (Table 1).

### 3.2. Procedure Characteristics

Table 2 shows the procedure characteristics. Resections were NCR in 19 patients (82.6%) and CR in another four (17.4%). 21 resections were judged curative (91.3%). In two patients (G3 sm1 L1 Rx at the VM and G3 sm1 L0 V0 R0, respectively), surgery was recommended but both patients refused. Both patients remained free of recurrence during follow-up of 40 months and 37 months, respectively.

### 3.3. Strictures

**Figure 2:**
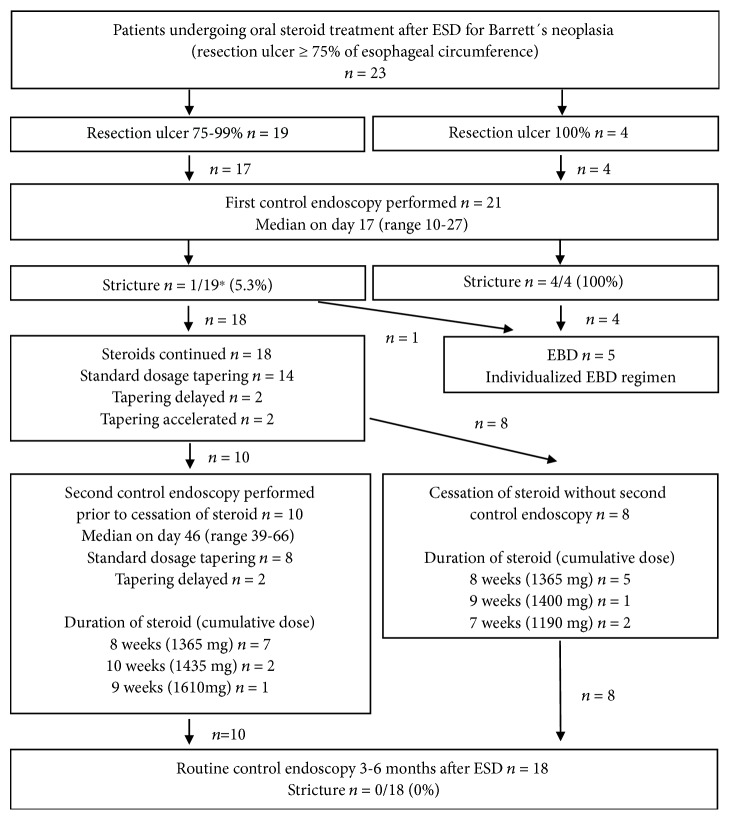
Clinical course of patients receiving oral prednisolone prophylaxis. EBD: endoscopic balloon dilatation. ^∗^The patient with the stricture had stopped steroid treatment without reasons and without side effects.

23 patients started oral steroid therapy on the day after ESD. Patients' course is shown in Figure 2. One patient stopped steroid treatment without reasons and without notable side effects on the fourth day after ESD. He refused a scheduled control endoscopy and presented with a symptomatic stricture on day 27. Stricture was treated with three sessions of EBD. 17/19 patients with NCR and all four patients with CR underwent the recommended first control endoscopy after 2-3 weeks. At that time, all patients with CR had developed symptomatic strictures despite continued daily prednisolone dose of 30-40 mg. Repeated EBD was performed (median 6.5 sessions; range 3-14 sessions for 8-40 weeks). The length of the resection ulcer was not significantly different between the NCR and CR groups (median 42.5 vs. 50 mm; *p* = 0.19). None of the patients who continued steroid prophylaxis after NCR had developed a stricture at first control endoscopy, and steroids were tapered according to the degree of reepithelialization of the ESD ulcer. In ten patients, second control endoscopy was performed before stopping the steroid therapy. In another eight patients, second control endoscopy was not performed in time because of patient's refusal. In these patients, prednisolone was stopped according to the standard tapering regimen. In summary, 12 patients received the standard prednisolone regimen over 8 weeks. Treatment duration was prolonged to nine weeks in two patients and to ten weeks in another two. Decision to delay the treatment was made after the first control endoscopy in two and after the second control in the other two. In two patients, treatment was shortened to seven weeks after the first control endoscopy. The first control endoscopy was delayed on days 23-27 in three patients. None of these patients needed modification of the treatment. One patient had refused any control endoscopy and completed the standard steroid regimen over 8 weeks without a stricture. In summary, no stricture was seen in the NCR group when steroid prophylaxis was completed. In contrast, the stricture rate was significantly higher in the CR group (100% vs. 5.3%; *p* < 0.001). None of the patients developed infections or other side effects during steroid treatment. Three patients had concomitant type 2 diabetes mellitus (DM) and used oral antidiabetics at the time of ESD. Adjustment of the antidiabetic medication was not needed in any of them. None of the patients without DM at the time of ESD developed DM during steroid treatment.

**Figure 3:**
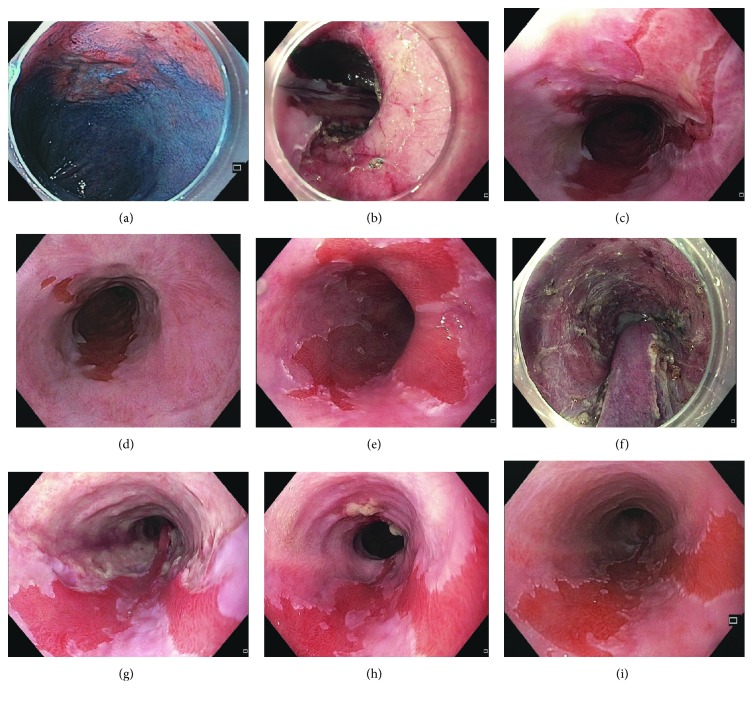
Examples for ESD and stricture prevention in large Barrett's neoplasia. (a) Early adenocarcinoma 40 mm in diameter within BE C7M8. (b) Resection ulcer after ESD involving 80% of the circumference. (c) First control endoscopy on d16 after ESD (prednisolone dose 30 mg): rapid reepithelialization, no stricture, mild dysphagia, and standard steroid tapering. (d) Second control endoscopy on d43 after ESD (prednisolone dose 10 mg): complete ulcer healing without stricture, standard steroid tapering (duration 8 weeks). (e) Multifocal early adenocarcinoma within BE C4M6. (f) Resection ulcer after ESD involving 90% of the circumference. (g) First control endoscopy on d12 after ESD (prednisolone dose 40 mg): no rapid reepithelialization, no stricture, no dysphagia, and standard steroid tapering. (h) Second control endoscopy on d47 after ESD (prednisolone dose 10 mg): residual ulcer without narrowing of the lumen. Prolongation of steroid tapering (duration 10 weeks). (i) Endoscopy on day 80 after ESD: complete ulcer healing without stricture.

Figure 3 shows examples of different courses after extensive ESD.

### 3.4. Other Complications

The bleeding rate was 4.3% (1/23). The patient presented with hematemesis 20 hours after circumferential ESD, and a small nonbleeding vessel was treated with endoscopic clip application. Blood transfusion was not indicated. No perforation- or procedure-related mortality was observed.

### 3.5. Follow-Up

In eight patients, complete elimination of BE was achieved with ESD. In one patient, a small metachronous mucosal cancer (diameter 10 mm) was resected with EMR six months after ESD, and residual Barrett's was ablated using radiofrequency ablation (RFA) another three months later. One patient, who had developed stricture after circumferential ESD, underwent repeated EBD for 6 months and refused RFA afterwards. Metachronous mucosal cancer (diameter 8 mm) was detected close to the stricture 20 months after ESD and repeated ESD was performed. Residual nonneoplastic Barrett's was treated with radiofrequency ablation in two patients, with APC in another three and with a combination of RFA and APC in another two patients.

None of the patients who received ablative therapies developed a stricture after ablation. One patient died six months after ESD because of metastatic renal cell carcinoma. Another patient died 12 months after ESD because of multiple myeloma. Median follow-up was 21 months (range 3-54).

## 4. Discussion

If ER exceeds 75% of the esophageal circumference, the risk for postinterventional stricture is reported to be as high as 66-100% [13–15]. Today, orally administered or locally injected steroids are first-line treatment options for stricture prevention [14, 15]. These techniques have been shown to reduce but not to eliminate the stricture risk, and the ideal treatment modality for stricture prevention remains undefined. Available data, published mainly by Asian authors, are restricted to ESD of superficial SCCs. In contrast, in Western countries, early SCC is rare and EAC arising within BE is the predominant indication for esophageal ER. EMR is the endoscopic resection method of choice for small Barrett's neoplasia and rarely causes strictures. Pech et al. reported 12 strictures in 1000 EMRs for early EAC [16]. ESD can be considered in lesions exceeding 15 mm, poor-lifting lesions, and lesions at risk for submucosal invasion [2]. Following this strategy, resections exceeding three quarters of the esophageal circumference are infrequent but unavoidable in some cases with large or multifocal neoplasia. In our study, 27/197 resections exceeded three quarters of the circumference (13.7%) and four resections were performed circumferentially (2.0%).

In 2015, we published our first data on ESD in early esophageal cancer which included nine EAC resections exceeding 75% of the circumference [6]. In the first six patients, prophylactic EBD was performed. Stricture developed in five of them (83.3%) and further EBD was required. During the later study period, three patients received a fixed-dose 8-week oral steroid prophylaxis according to the Japanese SCC data (starting with prednisolone 40 mg daily followed by a weekly reduction of 5 mg). However, two of them (66.7%) developed dysphagia during the steroid tapering period and EBD was required. In 2011, Yamaguchi et al. had reported a stricture rate of 5.3% after oral steroid prophylaxis for ESD exceeding three quarters of the esophageal circumference [8]. Isomoto et al. reported a 50% stricture rate after circumferential ESD using the same regimen [9]. In both studies, a fixed-dose prednisolone regimen was used after ESD for SCC without routine control endoscopies (starting with 30 mg daily and tapering 30/30/25/25/20/15/10/5 mg weekly over eight weeks). Kataoka et al. described a 17.6% stricture rate for a shortened prednisolone regimen (starting with 30 mg daily and weekly tapering 30/20/10 mg over three weeks) [10]. So far, only one retrospective study using steroids after EMR in BE is available. Ratone et al. reported a 13% stricture rate using Yamaguchi's regime in 31 patients. However, he included resection ulcers exceeding 50% that makes interpretation of the data difficult [17]. Today, local injection of triamcinolone into the resection ulcer immediately after ESD is the preferred treatment strategy in Asia. Local injection is preferred in order to avoid potential side effects of systemic steroid treatment. Hanaoka et al. could reach a 10% stricture rate after injecting 100 mg triamcinolone in the ESD ulcer (one injection, fixed dose) while Hashimoto et al. reported a 19% stricture rate after repeated triamcinolone injection (days 3, 7, and 10; dose 18-62 mg) [11, 12]. A Japanese prospective randomized control trial is ongoing to compare systemic prednisolone therapy (Yamaguchi's regime) and local triamcinolone injection (Hanaoka's regime) [18]. The results are awaited and the ideal treatment regime remains undefined today, especially in Barrett's resections.

During our first study, we had seen different courses of ulcer healing and stricture development during routine endoscopies in patients undergoing prophylactic EBD [6]. We proposed that the stricture risk could be minimized when the individual scarring process would be taken into account for tapering the steroid dose and when epithelialization of the resection area would be completed before stopping the steroids. Taking these considerations into account, we decided to use a steroid regimen with a higher starting dose (prednisolone 50 mg) and individualized tapering according to the individual ulcer healing process (assessed endoscopically during the first tapering period and before stopping the steroids). Using this strategy, we could avoid strictures in all patients with NCR. 95% of our patients underwent a first control endoscopy 2-3 weeks after ESD, and the steroid tapering was modified in 21% according to different courses of ulcer healing. It remains speculative if the higher steroid dose or the endoscopy-based individualization of the tapering regimen has influenced the stricture development. In particular, the role of the second control endoscopy which was not performed in most patients seems questionable.

In contrast to patients with NCR, all patients with CR developed a symptomatic stricture within the first 2-3 weeks and repeated EBD was required. The stricture risk after CR has been addressed in Asian publications on ESD of SCCs. Hanaoka et al. described a stricture in 11/12 patients treated with local triamcinolone and up to 40 sessions of EBD were required. CR was an independent risk factor for stricture in his study (adjusted OR 19.77; 95% CI 4.67-8.72) [19]. Recently, Iizuka et al. reported a modified oral steroid regimen starting with 30 mg prednisolone and reducing the daily dose by 5 mg every three weeks (resulting in a prolonged treatment duration of 18 weeks). However, 10/11 patients had received additional local triamcinolone injections. The stricture rate after CR of SCCs was 36.4% and significantly lower compared to 82% after using Yamaguchi's regimen over 8 weeks [19]. Potential side effects of oral steroid prophylaxis regimens are feared but discussed controversially. Using a fixed-dose oral regimen over 8 weeks, Yamaguchi et al. did not report any side effects in 22 patients [8]. In contrast, Iizuka et al. reported three infections when nine patients were treated with the same regimen (pneumonia, oral herpes infection) [19]. Ishida et al. reported a case with severe disseminated nocardiosis during oral steroid prophylaxis [20]. In our study, we could confirm Yamaguchi's data and did not find infectious complications or other serious side effects in any patient. Patients should be informed about potential side effects and should be monitored carefully during the steroid treatment. Sufficient data not only on stricture prevention but also on steroid side effects are awaited from the ongoing Japanese multicenter study [18].

Today, it remains unclear if the risk of postinterventional stricture development is different for SCC and EAC and if Asian results are transferable to Western countries where EACs represent the vast majority of esophageal lesions.

Limitations of the study are the retrospective design, the small patient number, and the missing control group. Some patients did not undergo the second control endoscopy which is another limitation. Randomized controlled trials comparing different strategies are needed to define the ideal prevention strategy after extensive ER in neoplastic BE.

## 5. Conclusion

In our small study, oral steroid administration with an endoscopy-based individualization of dosage and treatment duration was sufficient to prevent strictures after extensive but noncircumferential ER of EAC. The stricture rate was lower compared to all previous studies reporting on steroid prophylaxis [14, 15]. In contrast, strictures could not be avoided after circumferential resection. After circumferential resection of neoplastic Barrett's esophagus, EBD should be started early. Circumferential extension of EAC is a very rare scenario and CR should be restricted to these rare lesions. The strategy for stricture prevention after CR needs to be further improved.

## Figures and Tables

**Table 1:** Patients and lesion characteristics.

	*n* = 23
*Clinical characteristics*	
Age, median (range) (years)	67 (45-84)
Sex, male/female, *n*	21/2
ASA grade, 1/2/3, *n*	8/12/3
*Barrett's extent*	
C (median, range) (cm)	2 (0-9)
M (median, range) (cm)	5 (2-10)
Hiatal hernia, *n* (%)	21 (91.3)
*Lesion characteristics*	
Paris classification, *n* (%)	
0-Is	2 (8.7)
0-IIa	11 (47.8)
0-IIb	9 (39.1)
0-IIc	1 (4.3)
Endoscopic estimation of neoplasia	
Single lesion, *n* (%)	13 (56.5)
Estimated diameter of single lesion; median (range), mm	40 (20-60)
Multifocal neoplasia (≥1 visible lesion), *n* (%)	10 (43.5%)
Pretreated lesions	0

**Table 2:** Procedure characteristics (^∗^R0 for neoplasia was defined as R0 for cancer and high-grade dysplasia. ^∗∗^Rx resection was diagnosed at the HM in one lesion and at the HM in another).

Procedure time, median (range) (minutes)	150 (75-300)
*Resection rates,n(%)*	
En bloc resection	23 (100)
R status for neoplasia^∗^, R0/R1/Rx	21 (91.3)/0/2^∗∗^ (8.7)
R status for Barrett's metaplasia, R0/R1/Rx	8 (34.8)/15 (65.2)/0

*Resection ulcer*	
75-89% of the circumference, *n* (%)	12 (52.2)
90-99% of the circumference, *n* (%)	7 (30.4)
100% of the circumference, *n* (%)	4 (17.4)

*Resection specimen*	
Horizontal diameter, median (range) (mm)	70 (43-110)
Vertical diameter, median (range) (mm)	45 (20-65)

*Histopathological diagnosis*	
Adenocarcinoma, *n* (%)	23 (100)
Single lesion, *n* (%)	16 (69.6%)
Diameter of single lesion; median (range) (mm)	40 (10-60)
Multifocal neoplasia, *n* (%)	7 (30.4%)

*Histopathology,n*	
Invasion depth, mucosal (pT1a)/submucosal (pT1b)	21/2
Grading, G1/G2/G3	14/6/3
Lymphatic invasion	1
Vascular invasion	0

## Data Availability

The data used to support the findings of this study are available from the corresponding author upon request.
